# Shipping and the Spread of Infectious Salmon Anemia in Scottish Aquaculture

**DOI:** 10.3201/eid0801.010144

**Published:** 2002-01

**Authors:** Alexander G. Murray, Ronald J. Smith, Ronald M. Stagg

**Affiliations:** * Fisheries Research Services Marine Laboratory, Aberdeen, United Kingdom; † University of Aberdeen, Aberdeen, United Kingdom

**Keywords:** ships, epizootic, infectious salmon anemia virus, aquaculture, Scotland

## Abstract

Long-distance transport of pathogens plays a critical role in the emergence of novel diseases. Shipping is a major contributor to such transport, and the role of ships in spreading disease has been recognized for centuries. However, statistical confirmation of pathogen spread by shipping is usually impractical. We present evidence of invasive spread of infectious salmon anemia in the salmon farms of Scotland and demonstrate a link between vessel visits and farm contamination. The link is associated with vessels moving fish between sites and transporting harvest, but not with vessels delivering food or involved in other work.

## Human Development and Infectious Disease in Coastal Ecosystems

Anthropogenic activity increases the incidence of infectious diseases, which in turn influence the populations and production of marine organisms, from free-living bacteria (*1*) to mammals (*2*,*3*). The aquaculture industry has been strongly affected by diseases emerging from anthropogenic activities, and itself has played a critical role in their spread (*4*).

Infectious salmon anemia is an emerging disease causing severe damage to the salmon-farming industry in an increasing number of countries. The disease, first reported in Norway in 1984 (*5*), has since been reported in Atlantic Canada (1996) (*6*); Scotland (1998) (*7*); the Faroe Islands and possibly Chile (1999) (*8*); and most recently Maine, USA (late 2000) (*9*); over the last few months (2001), infectious salmon anemia has spread rapidly in Maine (*10*). In 1999, the annual cost of infectious salmon anemia was reported to be US$11M in Norway and US$14M in Canada, while in Scotland, the total cost of the epidemic of 1998-99 was US$32M (*11*). It is too early to say what the cost of the disease will be in Chile and the United States, but both countries have large salmon-farming industries. In almost all cases, infectious salmon anemia has mainly affected Atlantic salmon, *Salmo salar*, but in Chile, deaths have been reported among Coho salmon, *Oncorhynchus kisutch* (*8*). All deaths have been among farmed salmon.

Infectious salmon anemia is an emerging disease (*12*) caused by novel virulent strains of a virus that has adapted to intensive aquacultural practices and has exploited the associated traffic to spread both locally and internationally. Genetic (*13*,*14*) and phenotypic (*15*) differences suggest that this adaptation occurred independently in Europe and the Americas. The virus strains then aggressively expanded their geographic ranges.

Invading new areas is critical for the survival of exotic species (*16*), including pathogens (*12*,*17*). Shipping has been identified as a major factor in movement of exotic species to coastal regions (*16*,*18*). The role played by ships in the introduction and spread of Black Death (a virulent form of plague) in 14th-century Europe has been extensively chronicled (*19*). Recently, huge numbers of bacteria (8.3 x 10^8^ l^-1^), including *Vibrio cholerae,* the agent of cholera, and viruses (7.4 x 10^9^ l^-1^) have been detected in ballast water of ships entering U.S. waters (*17*). Given the increasing volume of shipping (*18*), introduction of pathogens to coastal ecosystems is likely to increase. The rapidly growing aquaculture industry, with its high densities of potential host monocultures, is based in such coastal ecosystems (*4*).

Numerical analysis of the role of shipping in spreading pathogens is usually not possible because of heavy ship traffic and the multitude of pathogen sources. In this article, we examine the role of shipping in the invasive spread of infectious salmon anemia among Scottish salmon farms.

## The Study

### *Infectious Salmon Anemia Virus* (ISAV) in Scotland

**Figure 1:**
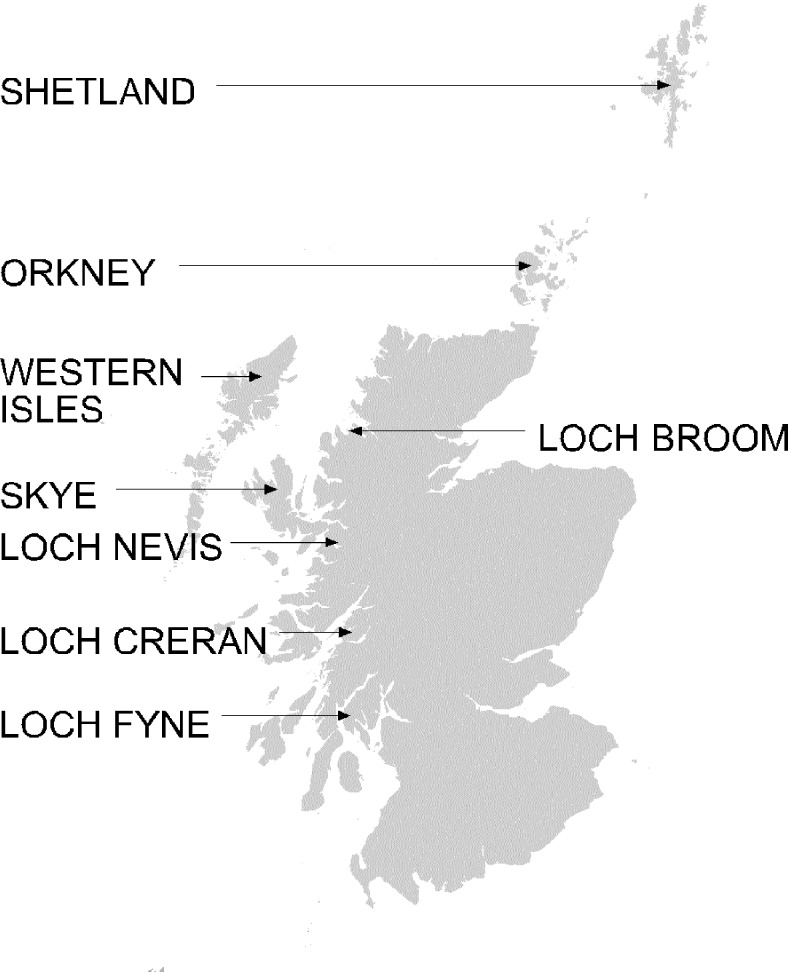
Map of Scotland showing locations named in text. The lochs shown are marine fjords.

In May 1998, the previously exotic viral disease infectious salmon anemia was detected at a salmon farm in Loch Nevis on the Scottish west coast (*7*,*20*,*21*) (Figure 1). ISAV is an othomyxovirus of a new genus (*Aquaorthomyxovirus*) that is closely related to the influenza viruses (*22*). ISAV subsequently spread to salmon farms throughout Scotland. Molecular epidemiologic studies indicate that a single strain was responsible for the initial epizootic (*14*), although a second strain unrelated to the outbreak was later identified (*23*). Infectious salmon anemia has been reportable in the United Kingdom since 1990 (*24*), so its spread has been well surveyed and documented. The pattern of spread was discontinuous, with farms in Shetland becoming infected during summer 1998 but ISAV detected in Orkney in late 1999. Many intervening areas did not become infected, and in many cases, farms close to infected sites did not become infected (*20*). The broad area of infection with multiple isolated foci is not consistent with diffusive spread of disease by fish-to-fish contact or through vectors such as parasites or seabirds (*25*,*26*); this is not to say that this form of spread may not have occurred occasionally or locally in the vicinity of outbreak foci.

Because of the broad pattern of spread, the movement of live fish and contaminated equipment between fish farms was suspected to play a role in the spread of this disease within Scotland (*20*). In aquaculture, salmon are moved extensively. They are reared at freshwater hatcheries, transferred to (and sometimes between) marine production sites, and finally transported to central harvesting and processing stations. In particular, well boats, which are used for the transport of fish, supplies, and equipment between farms and as work platforms, were suspected in the spread of ISAV (*20*).

Well boats and ferries have been suggested as possible routes of introduction of otherwise unexplained outbreaks of infectious salmon anemia in salmon farms in Norway, where the disease has been established since 1984 and has infected 101 fish farms in 1990 alone (*27*).

**Figure 2:**
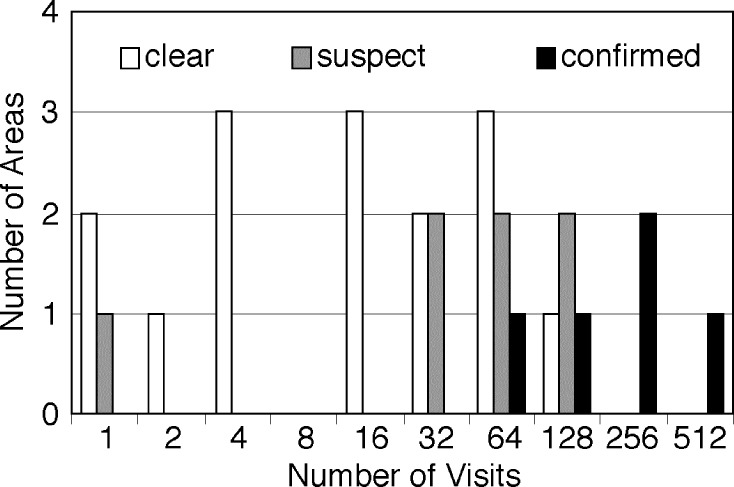
Distribution of infection by frequency of well-boat visits to salmon farms, Scotland.

We conducted a quantitative analysis of the spread of ISAV in Scotland and its relationship to the movements of four well boats that serviced the farmed-salmon harvesting center of Loch Creran in the year preceding the epidemic (May 1997 to May 1998). These well boats made 1,558 visits to fish farms along >850 km of coastline from Loch Fyne to Shetland. Farms not visited by these well boats are not included in this analysis (including in Orkney and the Western Isles, where ISAV was later detected). These farms were serviced by other well boats, for which movement data were not available. We grouped the visited farms into 26 areas since individual fish farms are not always identifiable from the boats’ logs. Of these areas, 6 were infected (*28*), 7 were suspected of being infected, and 13 escaped infection. Infectious salmon anemia was confirmed if clinical disease was present and ISAV was identified as the causal agent by both pathologic lesions and the presence of virus (*28*). In areas where infection was suspected, ISAV was identified by polymerase chain reaction (PCR) analysis (*29*) or similar immunofluorescence (*30*) methods or by clinical signs without confirmation of infection (*28*). A histogram of the number of areas included in these three categories clearly showed a relationship between the log number of boat visits and the risk for infection (Figure 2).

In two areas, infection developed after only a few visits. Loch Broom was suspected of being infected after a single visit by one of the boats. This infection occurred in 1999 and was therefore not part of the initial invasion. Infection also occurred on the Isle of Skye, where only two well-boat visits were recorded; however, in this case, fish stocks were moved directly from the infected site at Loch Nevis before infection was detected. Similarly, infectious salmon anemia was introduced to Shetland (*21*) when a partial load of smolts was brought to the Skye site, contaminating the load intended for shipment to Shetland. Since were were direct introductions of infected fish to these sites, we excluded them from most analyses.

The harvest-processing station at Loch Creran used by the four vessels is also excluded from further analysis. Visits to this processing station, where fish were unloaded and held temporarily in net pens before slaughter, were qualitatively different from harvesting visits to other sites, where fish were grown. The fish at Creran were from multiple sources, while fish at other sites were from one or a few sources. Because all harvest trips ended at the processing plant, its inclusion would lead to double counting of harvest trips. The exclusion of Creran is particularly important for analyzing the efficiency of infection transfer during different types of visits.

## Results

**Figure 3:**
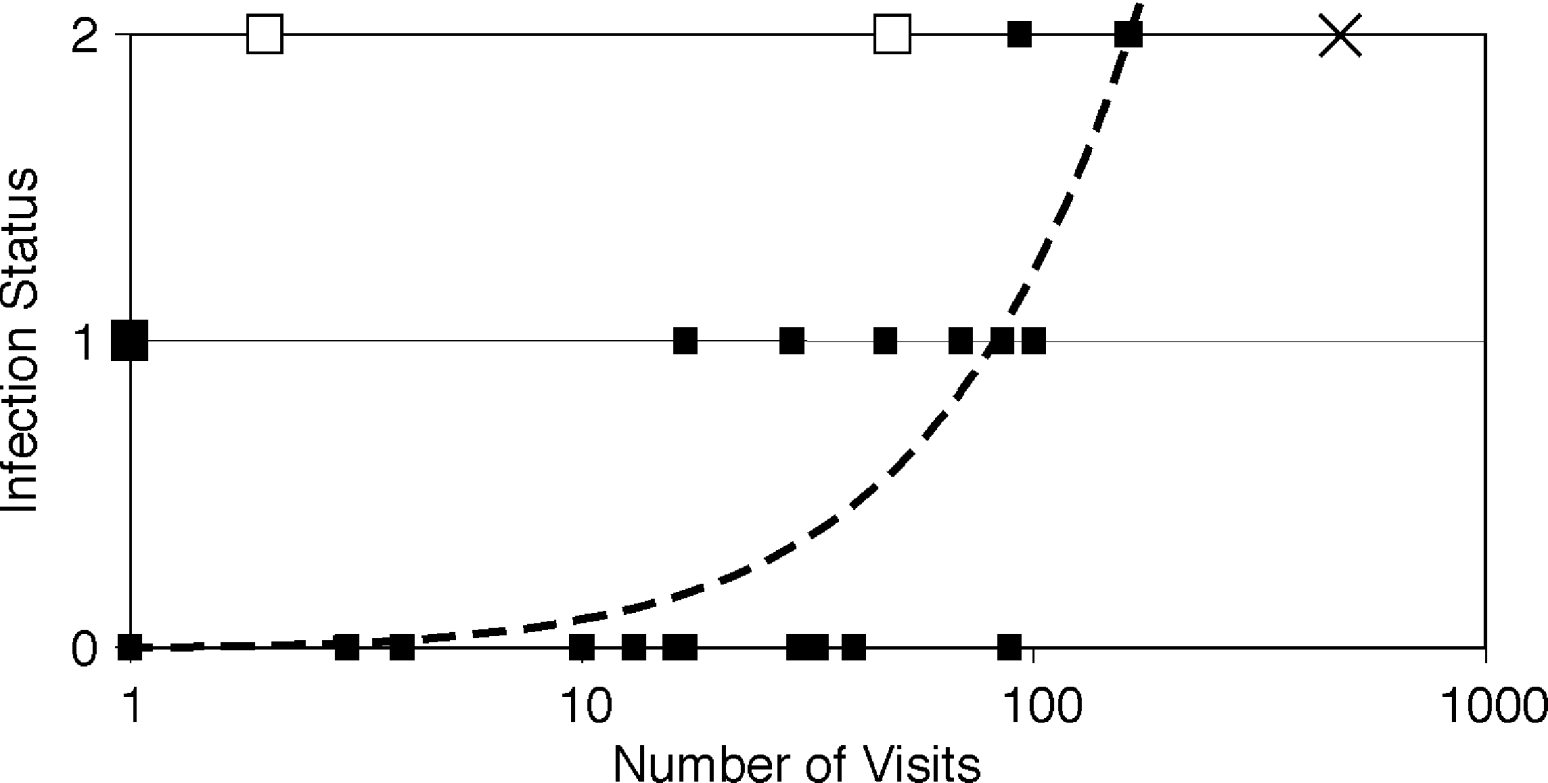
Infection status of areas versus number of well-boat visits. Infection status is 0 for no infection, 1 for suspected infection, and 2 for confirmed infection. Skye and Shetland, infected by fish transferred from Loch Nevis, are shown as large hollow squares, while the unconfirmed infection at Loch Broom is shown as a large triangle-filled square. Loch Creran is shown as a cross and is excluded to prevent double counting of harvest transport voyages. Regression is 0.012 x visits, r^2^ = 0.66, p = 0.000004.

The quantitative nature of the relationship between visits and infection status is determined by scoring areas that did not become infected as 0, areas with suspected infection as 1, and areas that did become infected as 2 (Figure 3). There is a significant relationship (regression of 0.010) between the number of visits and infection status (r^2^ = 0.23, p = 0.0015). However, if regions where infection is explained by the movement of infected fish (Skye and Shetland) and Lochs Broom and Creran are excluded, the relationship between number of visits and infection status becomes much more statistically significant (0.012 regression) (r^2^ = 0.66, p = 0.000004).

Well-boat logs divide vessel visits into three categories: shipment of harvest to Loch Creran, movement of live fish between other sites, and visits for general work plus delivery of food. We conducted a multivariate analysis of infection status versus these three categories of visits (Table); distance from Loch Creran is also included, showing that it is the number of these trips, not their length, that influences the spread of infection.

**Table:** Relationship between type and distance of visit by well boats and infection status of salmon farm, Scotland^a^

	Harvest	Fish movement	General	Distance
I: r^2^ = 0.43				
Relationship	0.011	0.021	0.0037	0.0006
p	0.03	0.09	0.65	0.59
II: r^2^ = 0.69				
Relationship	0.014	0.009	0.0057	-0.00096
p	0.001	0.43	0.28	0.29
III: r^2^ = 0.62				
Relationship	0.024	0.018	-0.0024	-0.010
p	0.05	0.41	0.82	0.44

^a^I: Excluding Creran only; II: excluding Creran, Loch Broom, Skye, and Shetland; III: within 50 km of Loch Creran (excluding Loch Creran).

The multivariate analysis shows that infection status is not related to distance from Loch Creran or movement of well boats for general purposes. Infection is transferred only by the shipment of live fish or visits to the harvesting site. If the sites infected by the movement of live fish from Loch Nevis and the uncertain site of Loch Broom are excluded, fish movements are also not related to infection status. Infected sites whose cause of infection is not explained by direct movement of infected fish are thus very strongly related to the number of harvest visits (p = 0.0009).

Harvesting involves transfer of live fish to Loch Creran, which is probably how this harvest-processing station became infected. At the time, the processing plant adjacent to the harvesting site was discharging effluent that had not been disinfected. Movement of the well boats from Loch Creran may then have rebroadcast ISAV.

Analysis of regional infection patterns within 50 km of Loch Creran shows a pattern of infection similar to the national pattern in that only movement of harvest vessels correlates with area infection status and r^2^ is similar at 0.62. However, the relationship is far weaker, only 12 points (p = 0.049), because fewer data are available. The regional pattern of infection excludes, among other areas, Skye, Shetland, and Loch Broom. At this regional scale, the processes and sensitivities of transmission do not appear to differ much from those at the national level.

At the smaller scale of spread of within a few kilometers, which the coarsely resolved available data cannot resolve, other processes may have become important, including physical transport of virus by currents (associated with waste products [*5**,**27*]), escaped or wild fish (*31*), or vectors (such as sea lice) (*32*) moving between neighboring farms. Infected wild salmonids, which have been found throughout Scotland (*33*) could act as an ISAV reservoir (*31*).

The salmon farming industry and regulatory authorities responded to the epizootic by introducing strict controls on hygiene and on movements of well boats and live and dead fish and by making the slaughter of infected stocks compulsory. These measures resulted in the closure of the Loch Creran site (among others) and control of the major route of infection. In 1999, sites not visited by these well boats (and also Loch Broom, which was reportedly only visited once) became ISAV suspect (*20*). It is possible that once ISAV had become widely distributed, vessels other than those based at Loch Creran transported ISAV to new locations. Other transmission processes (e.g., sharing divers or equipment) (*5*,*20*) could also have played a role in disseminating the virus.

## Conclusions

The evidence presented here supports a very strong quantitative link between the number of visits by well boats and the probability of ISAV detection in an area. The pattern of spread does not support a natural diffusive expansion of the ISAV epizootic, but the identical genetic nature of the ISAV at different sites indicates a direct link between incidents. Management activities could have resulted in interregional contact that coincided with the number of visits by well boats, but the strength of the relationship between well-boat visits and infection implies that the well boats played a predominant role in transmission. This role is emphasized by the relationships between the specific type of visit and infection status. Simple exchange of equipment does not appear to increase risk for ISAV infection in Norway (*5*), and neither do general well-boat visits in Scotland. We therefore conclude that it is the movement of well boats, through shipment of live fish and visits for harvesting, that spread ISAV at the regional and larger (ranging from 10 to several hundred kilometers) scale in Scotland during 1998-99.

No new incidents of clinical infectious salmon anemia occurred in salmon farms in Scotland in 2000, and infection did not develop in new areas, although more farms reported isolated ISAV-positive reverse transcription PCRs. ISAV also emerged in or was spread to the North Atlantic Faroe Islands. ISAV (but not infectious salmon anemia) was detected by PCR methods in wild salmonids throughout Scotland in 1999 (*33*), but although present, was much less prevalent in 2000 (*21*). The rapid response of the industry controlled the disease in farmed salmon and may have prevented endemic infectious salmon anemia in Scotland as it has in Norway (*5*,*27*).

After the introduction of stricter regulations in Norway in 1990 and 1991 to control hygiene in vessels and slaughterhouses, infectious salmon anemia outbreaks were reduced by nearly two orders of magnitude (*27*). This reduction shows that similar transport of poorly sanitized material by ships was also an important mechanism behind the infection’s spread in Norway. However, infectious salmon anemia is established in Norway, and since 1991, its incidence has gradually increased again.

Our data suggest that well boats have played a major role in the spread of infectious salmon anemia in both Scotland and Norway. The pathogens carried by well boats had two possible sources: the processing plant, when the wells were unloaded and replacement ballast water was contaminated with processing plant effluent, or the adjacent harvesting station, when infected water was taken up. Alternatively, infected fish or fish detritus may have remained in the wells or pumps and pipework after fish were discharged to the harvest station and the vessel immediately left for a harvesting operation at another site. When well boats transfer between sites, they are inspected and disinfected (*20*), which should minimize the risk for infected cargo residue transferring ISAV. However, removal of all fish and residue from pumps and pipework is problematic (*20*). At the time of the epidemic, effluent from the Loch Creran processing plant was not fully disinfected, so ISAV could have been present in ballast water taken up after disinfection. When the well boat starts harvesting, this water is discharged so that fish can be loaded. It is difficult to assess the role of the two sources of pathogens in the shipborne transmission of infectious salmon anemia, although since pathogens are abundant in ballast water (*17*), the role shown for the harvesting station in broadcasting ISAV could have wider implications for the transmission of diseases by shipping. Infectious salmon anemia is only one of many emerging diseases present in marine environments (*3*); the aquaculture industry can both suffer from and assist in the spread of such diseases (*4*). While individual movements of potentially infected fish, particularly live stocks (*34*), processed or unprocessed carcasses used for fish food (*3*), or fish imported for human consumption (*22*), are most likely to spread disease, these movements can be monitored and controlled. Extensive ship traffic (*18*) and lack of regulation increase the risk of spreading disease to animals raised for aquaculture and to other animals in marine environments (*4*).

Diseases potentially spread by shipping include waterborne diseases of humans such as cholera (*17*) and potential viral zoonoses (*35*). Thus, although this article highlights anthropogenic spread of a pathogen economically damaging for aquaculture, it also underscores the potential role of shipping in the global transport of zoonotic pathogens.
