# Correction, Vol. 8, No. 5

**DOI:** 10.3201/eid0809.020901

**Published:** 2002-09

In “Phylogenetic Analysis of a Human Isolate from the 2000 Israel *West Nile virus* Epidemic” by Thomas Briese et al., errors occurred in the text and figure legend. On page 529, right column, line 25, and in the figure legend on page 530, the host species for ISR-00PigC is pigeon. Additionally, in the figure legend, the GenBank accession no. for ISR-00PigC is AF380671, and the GenBank accession no. for WNV-ROM96(0334)-1996 is AF205879.

The online article at http://www.cdc.gov/ncidod/EID/vol8no5/01-0324.htm has been corrected.

We regret any confusion these errors may have caused.

